# Draft Genome Sequence of *Telmatospirillum siberiense* 26-4b1, an Acidotolerant Peatland Alphaproteobacterium Potentially Involved in Sulfur Cycling

**DOI:** 10.1128/genomeA.01524-17

**Published:** 2018-01-25

**Authors:** Bela Hausmann, Petra Pjevac, Katharina Schreck, Craig W. Herbold, Holger Daims, Michael Wagner, Alexander Loy

**Affiliations:** a Research Network Chemistry meets Microbiology, Division of Microbial Ecology, Department of Microbiology and Ecosystem Science, University of Vienna, Vienna, Austria

## Abstract

The facultative anaerobic chemoorganoheterotrophic alphaproteobacterium *Telmatospirillum siberiense* 26-4b1 was isolated from a Siberian peatland. We report here a 6.20-Mbp near-complete high-quality draft genome sequence of *T. siberiense* that reveals expected and novel metabolic potential for the genus *Telmatospirillum*, including genes for sulfur oxidation.

## GENOME ANNOUNCEMENT

All three validly described strains of *Telmatospirillum siberiense* were isolated from a mesotrophic Siberian peatland (1). In addition, closely related 16S rRNA gene sequences were recovered from other peatlands (2–8). Uncultured members of the genus *Telmatospirillum* have been associated with the anaerobic degradation of glucose (2), butyrate (8), acetate, propionate, and lactate (3, 7) in peat soils. Furthermore, in the literature, uncultured *Telmatospirillum* spp. were stimulated by propionate and butyrate under sulfate-reducing conditions (7), indicating a yet-unresolved role in sulfur cycling.

We obtained the draft genome sequence of *Telmatospirillum siberiense* 26-4b1 (DSM 18240), the type strain of the only validly described species of this genus (1). DNA was isolated using the DNeasy blood and tissue kit (Qiagen), and sequencing libraries were prepared using the Nextera XT kit (Illumina) and sequenced with the Illumina HiSeq 2000 platform. Raw reads were assembled using SPAdes (version 3.6.2) (9) and subsequently iteratively (*n* = 4) reassembled with SPAdes (version 3.11.1) using contigs >1 kb from the previous assembly as the “trusted contigs” input. The draft genome sequence consists of 81 scaffolds, with a total size of 6,202,994 bp, a G+C content of 62.3%, and an *N*_50_ value of 131,736 bp. Based on CheckM (10), the completeness of the draft genome is 99.5%. The genome was annotated using Rapid Annotation of microbial genomes using Subsystems Technology (RAST) (11) and the NCBI Prokaryotic Genome Annotation Pipeline. The draft genome contains 5,405 coding sequences (CDSs) and 48 tRNAs. Furthermore, the rRNA genes are carried on a small scaffold, with a coverage of 1,210×, although the average genome coverage was 216×, indicating the presence of 5 to 6 rRNA operons.

*T. siberiense* was reported to grow anaerobically, utilizing organic acids and sugars as energy and carbon sources, with the capability for nitrogen fixation. Autotrophic growth on hydrogen and tolerance to low oxygen pressure (up to 5 kPa) were also observed (1). As expected, the draft genome contains the genetic repertoire for these physiological traits. *T. siberiense* encodes the Embden-Meyerhof-Parnas (glycolysis) pathway, pentose phosphate pathway, Entner-Doudoroff pathway, and oxidative tricarboxylic acid cycle. It possesses genes of lactate dehydrogenases, for mixed acid fermentation, and several pathways for monosaccharide degradation. Three nitrogenase operons (two [FeMo]nitrogenases and one [FeFe]nitrogenase) and three [NiFe]hydrogenases (groups 1c, 1d, and 2b) (12) were identified. Respiratory complexes I to IV are present, including high-affinity terminal oxidases (cytochrome *bd* and *cbb*_3_ types) that enable the aerobic growth of *T. siberiense* at low oxygen concentrations. The motility of *T. siberiense* is explained by flagellar genes and autotrophic growth by genes encoding the complete Calvin-Benson-Bassham cycle. Acidotolerance (1) is reflected by the presence of genes coding for a potassium-transporting ATPase (*kdpABCD*) and a potassium uptake system (*ktrAB*) (13).

Surprisingly, the genome analysis revealed a possible metabolic potential of *T. siberiense* for sulfur oxidation. A partial thiosulfate-oxidizing machinery (*soxEFXYZA*) and the sulfur-shuttling system DsrEFH were identified. The partial *sox* operon is syntenic with the operon structure of the alphaproteobacterial thiosulfate oxidizer *Starkeya novella* (14), in which *soxBCD* is located separately. No other *sox* or *dsr* genes are present in the draft genome. Experimental evidence is needed to confirm a potential role of *T. siberiense* in sulfur cycling.

### Accession number(s).

The draft genome sequence of *Telmatospirillum siberiense* 26-4b1 was deposited in GenBank under the accession number PIUM00000000.
